# Cemeteries Are Effective Sites For Monitoring La Crosse Virus (LACv) and these Environments May Play a Role in LACv Infection

**DOI:** 10.1371/journal.pone.0122895

**Published:** 2015-04-10

**Authors:** Rebecca T. Trout Fryxell, Kimberly Freyman, Armando Ulloa, Brian Hendricks, Dave Paulsen, Agricola Odoi, Abelardo Moncayo

**Affiliations:** 1 Department of Entomology and Plant Pathology, University of Tennessee, Knoxville, Tennesee, United States of America; 2 Tennessee Department of Health, Nashville, Tennesee, United States of America; 3 Centro Regional de Investigación en Salud Publica, Instituto Nacional de Salud Pública, Tapachula, Chiapas, Mexico; 4 Department of Biomedical and Diagnostic Services, University of Tennessee, Knoxville, Tennesee, United States of America; University of California Davis, UNITED STATES

## Abstract

La Crosse encephalitis (LAC) is the leading arboviral disease among children, and was previously limited to the upper Midwest. In 2012 nine pediatric cases of LAC occurred in eastern Tennessee, including one fatal case. In an attempt to identify sites near an active LACv infection and describe the abundance and distribution of potential LACv vectors near a fatal LAC case in the Appalachian region, we initiated an end of season study using a combination of questing and oviposition mosquito traps placed at forty-nine sites consisting of cemeteries and houses within 16 radial kilometers of two pediatric infections. LACv was isolated from three *Aedes triseriatus* pools collected from cemeteries and spatial clustering analysis identified clusters of *Ae*. *triseriatus* and *Ae*. *albopictus* populations that overlapped in the same area as the 2012 LACv cases. Results indicate cemeteries are effective sites for monitoring LACv. The role of cemeteries and specific environmental features will be the focus of future investigations.

## Introduction

In North America, the leading arboviral disease among children is La Crosse encephalitis (LAC), and its incidence is growing around four geographic regions [1–3]. LAC has been limited to the upper midwest, but since 2003, the southern Appalachian region, Tennessee in particular, has the highest incidence risk in the nation: 228.7 cases per 100,000 children 15 years and younger, and almost 75% of all US cases reported in a year are in Appalachia [2], [4], [5]. The majority of LAC cases occur in children under 16 years, and present as a ‘summertime illnesses’ which often lead to summer diagnoses of meningitis [6–8].

La Crosse encephalitis virus (LACv) is now endemic in southern Appalachia [1–3] and is maintained in the environment through transovarial transmission (viral transmission from the female to her offspring) within the primary vector *Ae*. *triseriatus* [9] as well as through horizontal transmission of the vector biting its amplifying diurnal hosts (squirrels and chipmunks) [10–13]. Two additional vectors were introduced to southern Appalachia; *Ae*. *albopictus* in 1997 and *Ae*. *japonicus* in 2003 [3]. All three mosquitoes have been implicated as competent vectors of LACv, are commonly found in LACv endemic areas, and all are aggressive, diurnal, floodwater mosquitoes that oviposit desiccant-tolerant eggs in forest stands and opportunistically oviposit in artificial containers [12], [14]. Following the introduction of *Ae*. *albopictus* and *Ae*. *japonicus* to the southeastern region LAC cases increased, indicating a variation in virus and vector ecology as both invasive species will colonize with *Ae*. *triseriatus* disrupting the oviposition community. All three mosquitoes compete for similar mammalian hosts, and having all three live sympatrically increases the number of potential LACv vectors in a single area [15].

On July 11, 2012, a 6 year-old child presented to the emergency room after two seizures. On the second day of admission, the child displayed altered mental status, decreased mobility, hallucinations and vomiting. By day three, the patient was responsive only to painful stimuli and showed decreased arousal. The patient suffered another seizure on day four, was flaccid and unresponsive to stimuli with pupils nonresponsive to light. The patient progressively deteriorated and died on the fifth day after admission. Serum was collected for arboviral testing including West Nile, La Crosse, St. Louis, Eastern Equine and Western Equine viruses. Indirect immunoflurescent assay titers showed a four-fold increase in both IgM and IgG for LACv. LACv was subsequently isolated from brain tissue and confirmed via genome sequencing at CDC Fort Collins [16].

In an attempt to identify potential field sites after the LACv infection for the following year’s LACv ecology studies near the fatal case, we initiated an end of season field study. Since cemeteries have vases, established trees, are typically designed in a park-like / forested setting, and are open to the public, we hypothesized that cemeteries would be effective sites for monitoring LACv vectors. A combination of questing and oviposition mosquito traps were placed at forty-nine sites consisting of cemeteries and houses within 16 radial kilometers (ten miles) of two pediatric infections (including the deceased child) from September 5 to October 3, 2012. The objectives of the study were to identify sites near an active LACv infection and describe the abundance and distribution of potential LACv vectors near a fatal LAC case in the Appalachian region.

## Materials and Methods

### Experimental Design

**Fig 1:**
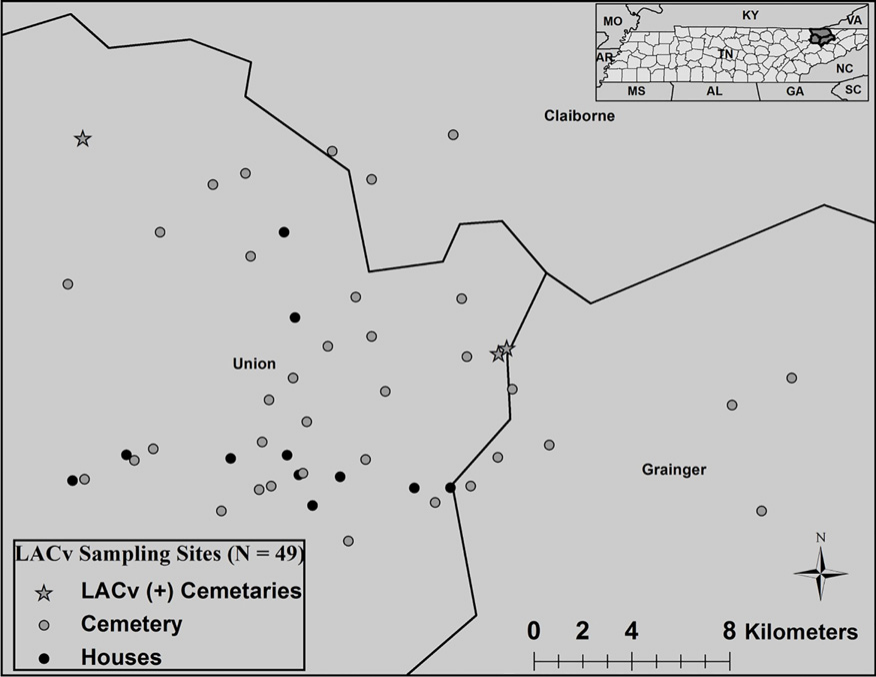
Three cemeteries were LACv positive. Forty-nine sampling sites consisting of private residential properties (n = 11; black circles) and publically accessible cemeteries (n = 38; gray circles) were sampled; three sites (gray stars) had mosquitoes positive with LACv.

Due to confidentiality issues, we did not collect mosquitoes at the 2012 LACv fatal case residence. Epidemiological investigations via the Tennessee Department of Health (TNDOH) identified the 2012 fatal case and a second ‘neighboring’ LACv hospitalized case, which did not leave the area within the previous weeks. This left us with a known area to conduct our study approximately 8 weeks after both hospitalizations; consequently, this study was conducted at the end of the 2012 mosquito season for one month. A combination of private residential properties (n = 11) and publicly accessible cemeteries (n = 38) were selected within 16 radial km (~10 miles) of the fatal case based on feasibility and accessibility, totaling 49 sites in eastern Tennessee U.S.A. consisting of Union, Grainger, and Claiborne counties (Fig 1). Eleven homes (residential sites) were included in the study of which one site was a previous 2012 LAC positive case residence that was hospitalized, a second residential site was the residence of a LAC negative case that was hospitalized in 2011, and nine additional residential sites were treated as unknowns because they had no previous report of LACv. The 38 cemetery sites were primarily small family owned cemeteries bordered by a combination of woodlands, rangelands, and urban development. All owners gave permission to access and use private lands. No specific permissions were required for activities at cemetery sites as all were open to the public.

### Residential Surveillance

At each residential site, adult mosquitoes were collected weekly from 5 Sept through 3 Oct 2012 using five standard traps. Two CDC questing traps were baited with ~2.3 kg of dry ice and the light was removed (model 512 John W. Hock, Gainesville, Florida), two BG sentinel traps baited with ~2.3 kg dry ice (model 2880 BG-Sentinel Trap, BioQuip Products, Rancho Dominguez, CA), and one CDC gravid trap baited with a grass-water infusion (model 1712, John W. Hock, Gainesville, Florida) operated overnight for a minimum of 14 hrs. Traps operated for 24hrs to capture diurnal, nocturnal, and crepuscular mosquitoes and were placed 20–100 m apart depending upon the site. To ensure viral RNA did not degrade, captured adult mosquitoes were kept alive as long as possible. For morphological identification to species and sex [17] mosquitoes were paralyzed with trimethylamine (mosquito and virus remained alive), and then stored at -80°C until screened for LACv. In addition, immature mosquitoes were collected with ovitraps baited with a week old grass-water infusion and lined with seed germination paper operated at each site for a week. Four ovitraps were placed at each of the nine unknown residential sites and five ovitraps were each placed at the previous 2011 LACv negative case and 2012 LACv positive case site; for a total of 46 ovitraps at residential sites. Weekly, ovitrap water was discarded and replaced and seed germination papers (termed egg papers) were replaced. Each egg paper was brought back to the laboratory and sent to the TNDOH for immature mosquito rearing, identification, and LACv screening.

### Cemetery Surveillance

In addition to the 11 residential sites, 38 cemeteries were selected for an in-depth ovitrap study. As with the house ovitraps, egg papers and water were replaced weekly, from 5 Sept.–3 Oct. 2012, this yielded a total of 760 egg papers. During the first two weeks of the study, two ovitraps operated at each cemetery site, and starting the third week (19 September) four ovitraps operated for the remainder of the study. All ovitraps were baited with water and egg paper as previously described, and placed at the four cardinal directions. Recovered egg papers were brought back to the laboratory and sent to the TNDOH for immature mosquito rearing as described above.

### Immature mosquito rearing and identification

A total of 816 egg papers (99.3% recovery) were collected and upon arrival at the TNDOH, all eggs on every egg paper were counted. Papers with more than 100 eggs were flooded first, followed by papers with less than 100 eggs. Each paper was flooded three times. Briefly, egg papers were dried and allowed to embryonate for at least one week in a humidified container. Each egg paper was placed in plastic trays (28x11x12 cm) containing 0.5–1 liters of deionized water and fed bovine liver powder ad libitum (#02900396 MP Biomedicals, Solon, Ohio). Every tray was placed into an incubator (60–80% relative humidity with a temperature of 26 ± 2°C, and a 12 h light 12 h dark photoperiod). Pupae of each tray were collected using plastic pipettes and transferred into small bowls in 60-cc screened cages for adult emergence containing 0.1 liter of water DI. Adults were removed using hand—held insect vacuums and placed in another chamber, frozen, and then morphologically identified to species and sex as described above [17].

### LACv Screening

Adults identified from the field or reared from egg papers were pooled into cohorts of ≤ 25 specimens of the same species, same sex, same trap, same date, and same site. To each mosquito pool, three copper BBs and 1ml of Eagle’s Minimum Essential Medium (2% FBS, 0.5% Na_2_Co_3_, and 1% antibiotics) were added. The samples were homogenized on a Retsch MM300 shaker for 90 sec, centrifuged at 5000 rpm for 5 minutes, and stored at -80°C until screened for LACv. 200 μl of resulting supernatant from homogenized mosquitoes was removed and RNA was purified using the QIAamp Viral Isolation 96 well protocol on the BioRobot 9604 or the QIAamp Viral RNA mini kit (Qiagen, Valencia, California). From the pooled RNA, 5 μl were used to screen for the presence of virus by reverse transcription PCR [18–20]. The initial reaction amplified a 251 bp piece of the S segment of the virus and was chosen for its ability to detect 24 different viruses in the Bunyamwera and California serogroups including LACv [18]. The second reaction amplified the M segment polyprotein genes as previously published [19], [20].

### Temporal and Spatial Analyses

All sites and traps were geocoded in ArcMAP 10.0 (Environmental Systems Resource Institute, ArcMap 10.0 ESRI Redlands, California) for spatial visualization and modeling. Since the numbers of houses and cemeteries were not equivalent, trapping at residential sites included both adult and immature collections while cemetery collections included only immature collections, and because cemetery sites were distinctly different from the residential sites (i.e., no structures, more vases, smaller and more forested area), statistical comparisons were limited. Additionally, since collections at houses were not standardized or replicated (i.e., different number of ovitraps per house), comparisons within site types were not conducted.

Due to residential site confidentiality, only cemetery traps were analyzed for evidence of spatial clustering. A spatial scan statistic, implemented in SaTScan [21], was used to investigate and identify clusters of *Ae*. *triseriatus*, *Ae*. *albopictus* and *Ae*. *japonicus* occurrence. The spatial scan statistic uses a circular window of variable radius that moves across the map. The radius of the window varies from 0 to a specified maximum value. As the window of the statistic moves across the map, it defines a set of different neighboring sampling sites. The radius of the circular scanning window was specified to vary from 0 to a maximum size that would include 50% of the vector population as per the recommendations of the software developer [22]. Thus, the specification of the scanning window size was based on the population (number of mosquitoes included in the analysis) and not on geographic distance. The cluster assessment was performed by comparing the number of mosquitoes within the window with the number expected if mosquitoes are randomly distributed in space. The test of significance of the identified clusters was based on a likelihood ratio test [23] the p-value of which is obtained by performing Monte Carlo testing. Identification of clusters of each of the three mosquito species was performed using Bernoulli probability model. For statistical inference, 999 Monte Carlo replications were performed. The null hypothesis of no clusters was rejected when the simulated p-value ≥ 0.05. The identified clusters were displayed cartographically using ArcView GIS 10 (Environmental Systems Resource Institute, ArcMap 10.0 ESRI Redlands, California).

Chi-square tests were performed to compare mosquito composition between sites (residential sites vs cemetery sites) and LACv positive and negative counts in this study to other studies [24] and p-values were considered significant when they were ≤ 0.05. The minimum infection rate (MIR) was calculated for the area and compared to previously published MIRs [24].

## Results

### LACv Results

A total of 6912 mosquitoes representing eight mosquito species were collected and they consisted of 5260 *Ae*. *triseriatus* specimens (76.1%), 1490 *Ae*. *albopictus* specimens (21.6%), 76 *Ae*. *japonicus* specimens (1.1%),44 *Culex erracticus* specimens (0.60%), 19 *Anopheles punctipennis* specimens (0.30%), 18 *Cx*. *pipiens-quinquefasciatus* specimens (0.30%), 2 *Ae*. *vexans* specimens (0.03%), 2 *An*. *quadrimaculatus* specimens (0.03%), and 1 unknown specimen (0.01%). This resulted in 782 mosquito pools that were all screened for LACv of which only seven were RT-PCR positive. All seven pools were *Ae*. *triseriatus* consisting of five male pools and two female pools, and all were from ovitrap papers collected at cemetery sites (Fig 1). RT-PCR positive samples had CT values ranged from 19.9367 to 36.3834 with a mean of 25.3113; no efforts were made to culture the virus. The first LACv positive pool was from a cemetery in the northern boundary of the study and consisted of two males that were collected 13 September 2012. Five of the pools came from the same ovitrap within a cemetery collected 3 October 2012, and the final pool was from a nearby cemetery on the same date. Taking into account only the *Ae*. *triseriatus* reared from cemetery egg papers, the MIR of 1.331 (7 LACv positive pools / 5260 *Ae*. *triseriatus* x 1000) was significantly greater than previous endemic reports a MIR of 0.26 in western North Carolina [23](*X* ^*2*^ = 70.61; df = 1; *P* <0.001).

### Residential Surveillance

None of the 141 adult mosquitoes collected from the residential sites were LACv positive. They consisted of *Ae*. *albopictus* (n = 46), *Cx*. *erraticus* (n = 44), *An*. *punctipennis* (n = 19), *Cx*. *pipiens-quinquefasciatus* (n = 18), *Ae*. *triseriatus* (n = 9), *Ae*. *vexans* (n = 2), *An*. *quadrimaculatus* (n = 2), and 1 unidentifiable specimen (Table 1). The 2012 positive case house had questing *Ae*. *albopictus* (n = 26) and *Ae*. *triseriatus* (n = 3); whereas, the LACv negative residential site had few questing LACv vectors; only two *Ae*. *albopictus* and zero *Ae*. *triseriatus*. LACv vectors collected from the nine remaining residential sites consisted of a total of 18 *Ae*. *albopictus* and 6 *Ae*. *triseriatus*. Adult collections from the two BG and two CDC traps at residential sites were comprised of 36% *Ae*. *albopictus*, 29% *Cx*. *erraticus*, 13% *An*. *punctipennis*, 9% *Cx*. *pipiens*, 8% *Ae*. *triseriatus*, 2% *Ae*. *vexans*, 2% *An*. *quadrimaculatus* and 1% of unknown species.

**Table 1:** Adult mosquitoes collected at residential sites during the three-week period.

Mosquitoes Species	5–6 Sept 1 CDC trap / site	12–13 Sept 2 CDC traps / site	19–20 Sept 2 CDC traps / site	Total
**2012 LAC Positive Site (n = 1)**				
*Ae*. *albopictus*	1	15	10	26
*Ae*. *triseriatus*	0	3	0	3
*Ae*. *vexans*	0	0	0	0
*An*. *punctipennis*	0	0	0	0
*An*. *quadrimaculatus*	0	0	0	0
*Cx*. *erraticus*	0	0	0	0
*Cx*. *pipiens-quinquefasciatus*	0	1	0	1
unknown	0	0	0	0
Total	1	19	10	30
**2011 LAC Negative Site (n = 1)**				
*Ae*. *albopictus*	0	1	1	2
*Ae*. *triseriatus*	0	0	0	0
*Ae*. *vexans*	0	0	0	0
*An*. *punctipennis*	0	0	0	0
*An*. *quadrimaculatus*	0	0	0	0
*Cx*. *erraticus*	0	0	0	0
*Cx*. *pipiens-quinquefasciatus*	0	0	0	0
unknown	0	1	0	1
Total	0	2	1	3
**LAC Unknown Sites (n = 9)**				
*Ae*. *albopictus*	8	7	3	18
*Ae*. *triseriatus*	0	5	1	6
*Ae*. *vexans*	0	2	0	2
*An*. *punctipennis*	6	11	2	19
*An*. *quadrimaculatus*	0	0	2	2
*Cx*. *erraticus*	14	30	0	44
*Cx*. *pipiens-quinquefasciatus*	10	7	0	17
unknown	0	0	0	0
Total	38	62	8	108
**All Residential Sites**				
*Ae*. *albopictus*	9	23	14	46
*Ae*. *triseriatus*	0	8	1	9
*Ae*. *vexans*	0	2	0	2
*An*. *punctipennis*	6	11	2	19
*An*. *quadrimaculatus*	0	0	2	2
*Cx*. *erraticus*	14	30	0	44
*Cx*. *pipiens-quinquefasciatus*	10	8	0	18
unknown	0	1	0	1
Total	39	83	19	141

**Fig 2:**
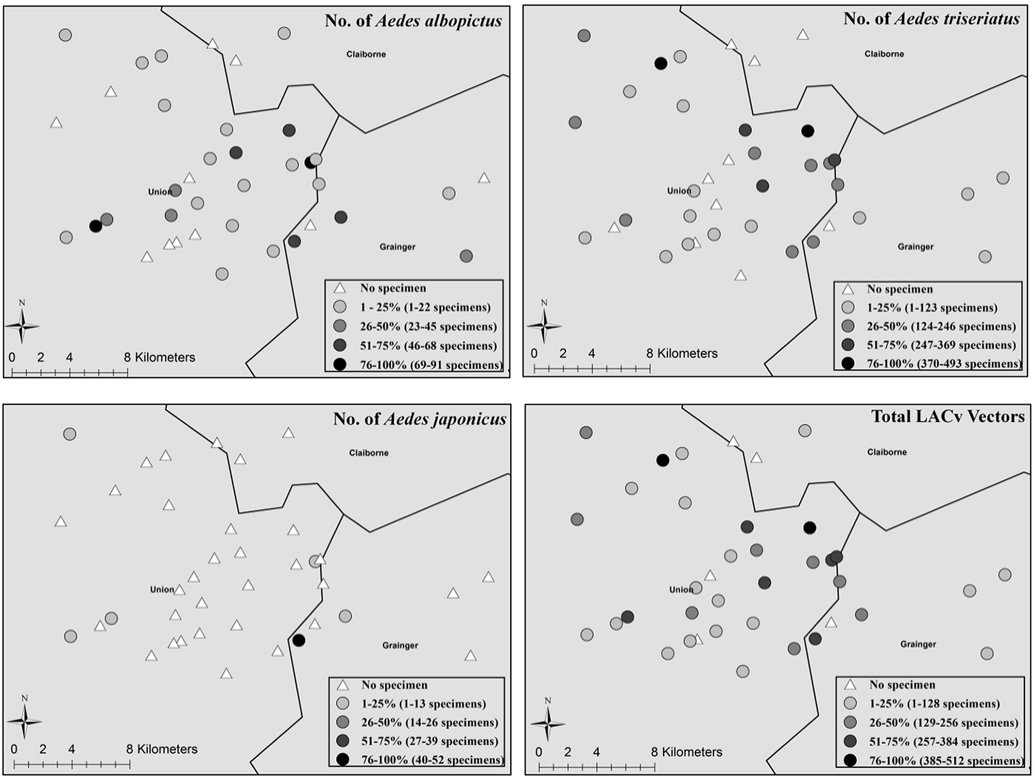
LACv vector populations. LACv vector populations varied across the entire study area; presented are the number of *Aedes albopictus* (A), *Ae*. *triseriatus* (B), *Ae*. *japonicus* (C), and all LACv vectors (D).

None of the 1847 mosquitoes raised from egg papers collected from residential sites were LACv positive. The mosquito species composition was *Ae*. *triseriatus* (58.1%, n = 1073 specimens), *Ae*. *albopictus* (41.4%, n = 765 specimens) and *Ae*. *japonicus* (0.5%, n = 9 specimens) (Fig 2). From the residential sites, 12.5% of the ovitraps had only one LACv vector species, 50% of the ovitraps had two species, and 37.5% had all three LACv vector species. The mosquito composition of ovitrap collections at each residential site differed slightly. The 2012 positive case house only had *Ae*. *triseriatus* mosquitoes reared from egg papers (n = 186; 46.5 ± 35.25 per week). The 2011 LACv negative residential site had 150 *Ae*. *albopictus* (all collected the week of Oct 3 from five egg papers) and 3 *Ae*. *triseriatus* (one collected the 12 Sept and two collected the 3^rd^ Sept). Ovitraps from the nine remaining residential sites yielded a total of 1508 mosquitoes (33.51 ± 8.75 mosquitoes per site per week), and these consisted of 884 *Ae*. *triseriatus* (19.64 ± 5.43 per site per week), 615 *Ae*. *albopictus* (13.67 ± 5.2 per site per week), and 9 *Ae*. *japonicus* (0.2 ± 0.16 per site per week). Descriptive statistics for each species collected with ovitraps at each residential site are presented in Table 2.

**Table 2:** Descriptive statistics of the three LACv vectors collected with ovitraps during the study.

Site (n = no. of egg papers)	*Aedes albopictus*		*Aedes japonicus*		*Aedes triseriatus*	
	total (% freq.)	mean (± SEM)	total (% freq.)	mean (± SEM)	total (% freq.)	mean (± SEM)
2012 LACv positive (n = 13)	0 (0%)	0	0 (0%)	0	186 (100%)	46.5 (± 2.405)
2011 LACv negative (n = 16)	150 (98.0%)	37.5 (± 37.5)	0 (0%)	0	3 (2%)	0.75 (± 0.479)
LACv unknown (n = 88)	615 (40.8%)	13.67 (± 5.186)	9 (0.6%)	0.2 (± 0.158)	884 (58.6%)	19.64 (± 5.432)
Residential Sites (n = 117)	765 (41.4%)	14.43 (± 5.151)	9 (0.6%)	0.170 (± 0.134)	1073 (58.1%)	20.25 (± 5.307)
Cemetery Sites (n = 154)	679 (13.8%)	3.59 (± 0.808)	67 (1.4%)	0.35 (± 0.278)	4178 (84.8%)	22.11 (± 3.271)
**TOTAL (n = 271)**	1444 (21.3%)	5.97 (± 1.317)	76 (1.1%)	0.31 (± 0.219)	5251 (77.6%)	21.70 (± 2.802)

### Cemetery Surveillance

**Fig 3:**
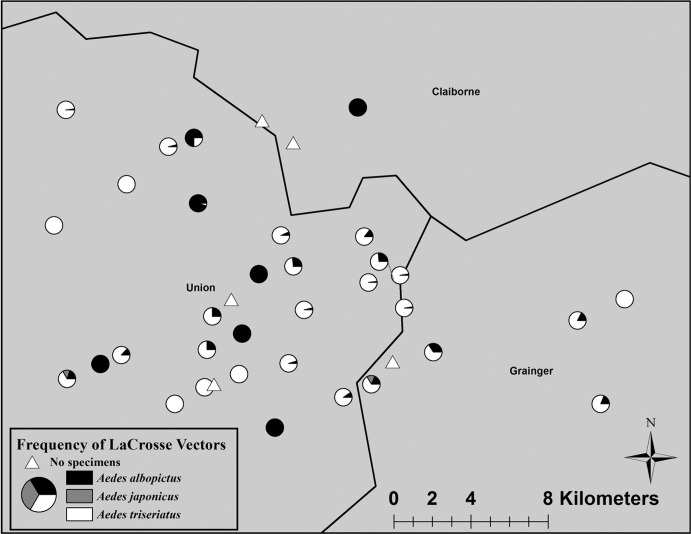
LACv vector compositions. Sampling from cemeteries allowed for the collection of *Ae*. *albopictus* (black), *Ae*. *japonicus* (light gray), and *Ae*. *triseriatus* (white). Only five cemeteries did not have any mosquitoes successfully rear to adults (small white triangle, some are hidden beneath collections).

While 19,779 eggs were counted from 610 egg papers (32.42 ± 2.46 eggs per egg paper), only 4,924 mosquitoes were reared and identified resulting in a 24.90% emergence rate (number of reared adults divided by the number of oviposited eggs). The reared mosquitoes consisted of 4,178 (84.8%) *Ae*. *triseriatus*, 679 (13.8%) *Ae*. *albopictus*, and 67 (1.4%) *Ae*. *japonicus* (Table 2). The composition of adults reared from each egg paper were primarily one species (189 occurrences) compared to 32 egg papers with two species and three egg papers with all three species. There were 316 incidences of no oviposition on egg papers, resulting in a patchy or pulse egg data (range of 0–475 eggs collected) as well as adult emergence data (0–254 adults reared). Species composition varied such that 108 egg papers had no eggs, 48 egg papers had one species emerge (34 had only *Ae*. *triseriatus* and 14 had only *Ae*. *albopictus*), 30 egg papers had two species emerge (27 *Ae*. *albopictus* and *Ae*. *triseriatus* papers, 2 *Ae*. *triseriatus* and *Ae*. *japonicus* papers, and 1 *Ae*. *albopictus* and *Ae*. *japonicus* papers) and 3 egg papers had all three species emerge. Cemetery mosquito populations varied across all sites (Fig 2) such that 27% of the sites had one species, 57.7% had two species, and 11.5% of the sites had all three LACv vector species. Five cemetery sites did not have any eggs collected in the ovitraps (Fig 3). The proportion of each vector reared from ovitraps was significantly different between cemeteries and houses (*X* ^*2*^ = 716.15; df = 2; *P* <0.0001).

### Spatial Clusters of Vector Distribution

**Fig 4:**
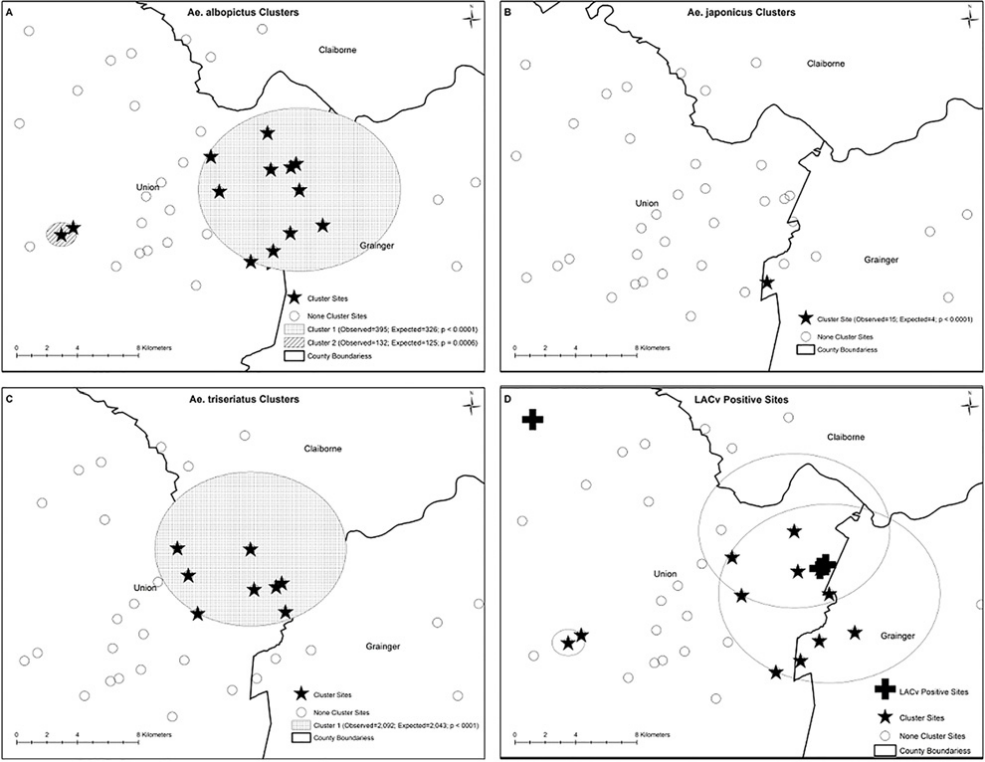
LACV vector spatial clustering analyses. Spatial clustering analysis indicated *Ae*. *albopictus* had two clusters (A), *Ae*. *japonicus* was not clustered (B), and *Ae*. *triseriatus* had one cluster (C). Of interest, the intersection of the *Ae*. *triseriatus* and *Ae*. *albopictus* clusters was in the vicinity of the 2012 LACv positive house and the 2012 LACv fatal case and near the LACv positive cemeteries (D).

Both *Ae*. *albopictus* (Fig 4A) and *Ae*. *triseriatus* (Fig 4C) showed evidence of significant spatial clustering. Two statistically significant high-risk clusters of *Ae*. *albopictus* were identified. The primary significant (*P* <0.0001) cluster had a total of 361 mosquitoes when only 325 mosquitoes should have been expected had they been randomly distributed in space. This cluster was comprised of a total of 11 sampled sites. The second much smaller *Ae*. *albopictus* significant (*P* <0.0006) cluster, included only two sampled sites and had a total of 132 mosquitoes when only 125 were expected had the mosquitoes been randomly distributed in space. *Aedes triseriatus* had the largest numbers with the single significant cluster (*P* <0.0001) reporting as many as 2,092 mosquitoes when 2,043 was expected. A very small, but significant (*P* <0.0001) cluster of *Ae*. *japonicus* was also observed (Fig 4B) and it had 15 mosquitoes when only four were expected had the mosquitoes been randomly distributed. The geographical areas of intersection between *Ae*. *triseriatus* and *Ae*. *albopictus* clusters covered the area where the two 2012 LACv cases were reported and included two of the three LACv positive cemeteries (Fig 4D).

## Discussion

Of the eight mosquito species collected only *Ae*. *triseriatus* was positive for LACv during this sampling period (all specimens and species were tested). Although pools of *Ae*. *albopictus* and *Ae*. *japonicus* did not test positive during this in-depth late-season sampling period, these species tested positive in other studies [1], [12], [14]. The period between the clinical presentation of LACv and our mosquito sampling was 56 days apart. This lag in clinical presentation and vector incidence may have accounted for the reduced mosquito infection rates since temperatures began to drop and all of these vectors were likely entering diapause. Previous sampling in June and July in other eastern Tennessee counties and western North Carolina counties identified LACv in *Ae*. *albopictus* and *Ae*. *japonicus* in June and July months [14], [24]; concurrent with the suggested time when the child in this study presented with LACv (July) and all three vector species are typically active and aggressive [25].

No direct comparisons were made between residential sites and cemetery sites due to differences in study and sampling design. In this study, residential ovitrap collections were distributed almost equally between *Ae*. *triseriatus* (58.1%) and *Ae*. *albopictus* (41.4%), while cemetery collections consisted primarily of *Ae*. *triseriatus* (84.8%). This finding suggests that an interspecies effect (e.g., competition) or an oviposition effect (e.g., oviposition choice) is occurring because only three egg papers (out of 540 egg papers of which 224 had eggs) had all three vectors reared and identified together. To confirm this, in-depth studies should be conducted to evaluate the interactions of the three vectors, determine the roles of each potential vector by identifying amplifying or maintaining vectors, and the role dispersal has in moving the virus from tree-holes to the urban environment. Additionally, the large previously unnoticed mosquito population at cemeteries suggests that cemeteries are effective and efficient sites for monitoring LACv in mosquito populations. Additional studies using these and similar cemetery sites could indicate that these sites (and those similar) serve as a bridge habitat between rural naïve sites and allow vector (and virus) populations to amplify and spread to the urban environments in east Tennessee. Future research should involve monitoring these populations as they may serve as predictors for future LACv outbreaks. The role of each vector and each habitat should be further investigated, especially since spatial clustering evidence around the case houses suggests both species (*Ae*. *triseriatus* and *Ae*. *albopictus*) overlapped with two 2012 LACv infections (Fig 4D). The biological relevance of the identified spatial clusters of high mosquito densities is that they provide useful information to guide future studies to investigate factors responsible for the identified significantly higher mosquito densities compared to areas that were not part of high mosquito density clusters. Thus, although a simple comparison of the number of observed vs expected vectors within a cluster may seem small (e.g. 2092 vs 2043), comparing the observed numbers in areas within a high density cluster compared to areas outside the clusters may be significant biologically and would be beneficial in guiding the geographical focus of future studies investigating reasons for observed spatial patterns. This would in effect provide clues to guide control efforts and future research. Identifying the environmental variables that make these overlapping areas and positive sites unique should be further studied.

Cemeteries were effective sites for monitoring LACv vector populations and conducting surveillance for LACv [26]. Of the 38 cemeteries, five of them did not have any viable eggs rear to adults and an additional five had less than ten adults emerge from the ovitaps. The three cemeteries that did have LACv positive mosquitoes had a minimum of 20 *Ae*. *triseriatus* collected during the positive week indicating these sites would permit LACv maintenance via transovarial transmission. Follow up work using cemeteries for surveillance and monitoring could prove useful as cemeteries are found throughout the Appalachian region, most are easily accessible, and they provide ideal habitats to a number of LACv reservoirs and vectors.

Within each cemetery, the authors noticed differences within mosquito oviposition behavior. While four ovitraps were placed at the different cardinal directions of each cemetery, certain ovitraps collected more mosquitoes than others suggesting some inherent differences within the relatively small area of a cemetery. The reasons for these differences remain unknown, but could be due to intra- or inter- species competition [27–29], the environment [1], [3], [30], the oviposition substrate [31–33], oviposition choice [34], [35], individual or species specific phenotypic or genotypic variation [36], [37], or random chance. Identifying the reasons for these differences could prove useful in LACv management.

The recent emergence and subsequent increased incidence risk of LACV in the Appalachian region coinciding with the introduction of *Ae*. *albopictus* and *Ae*. *japonicus* provides additional reason to suspect multiple vectors may be involved in LACv transmission. La Crosse encephalitis is the leading cause of arboviral disease among children in North America, and it is now endemic to the Appalachian region where approximately 75% of all US cases occur [38]. The reasons for these clustered incidences of LACv are currently unknown. The vectors and reservoirs of LACv have been documented, but still much research is needed to determine the mechanism of LACv transmission and identify ways to prevent childhood cases of La Crosse encephalitis. In 2013, 21 Tennessee children were diagnosed out of 74 reported cases in the U.S. justifying additional LACv research in Tennessee [39]. Here we report that cemeteries in an endemic area are effective sites for monitoring LACv and its vectors because all three vectors were reported in similar compositions to the 2012 LACv infected house and LACv was identified within the collection.

LACv has a patchy distribution and low prevalence rate so it is essential to maximize collection opportunities in research studies; data here indicates cemeteries are useful as LACv monitoring and surveillance sites as these are generally public property and accessible presenting less logistical issues for surveillance than private properties.
